# Asymptomatic infection of the fungal pathogen *Batrachochytrium salamandrivorans* in captivity

**DOI:** 10.1038/s41598-018-30240-z

**Published:** 2018-08-06

**Authors:** Joana Sabino-Pinto, Michael Veith, Miguel Vences, Sebastian Steinfartz

**Affiliations:** 1 Zoological Institute, Technical University of Braunschweig, Braunschweig, Germany; 2 Department of Biogeography, Trier University, Trier, Germany

## Abstract

One of the most important factors driving amphibian declines worldwide is the infectious disease, chytridiomycosis. Two fungi have been associated with this disease, *Batrachochytrium dendrobatidis* and *B. salamandrivorans* (*Bsal*). The latter has recently driven *Salamandra salamandra* populations to extirpation in parts of the Netherlands, and Belgium, and potentially also in Germany. *Bsal* has been detected in the pet trade, which has been hypothesized to be the pathway by which it reached Europe, and which may continuously contribute to its spread. In the present study, 918 amphibians belonging to 20 captive collections in Germany and Sweden were sampled to explore the extent of *Bsal* presence in captivity. The fungus was detected by quantitative Polymerase Chain Reaction (qPCR) in ten collections, nine of which lacked clinical symptoms. 23 positives were confirmed by independent processing of duplicate swabs, which were analysed in a separate laboratory, and/or by sequencing ITS and 28 S gene segments. These asymptomatic positives highlight the possibility of *Bsal* being widespread in captive collections, and is of high conservation concern. This finding may increase the likelihood of the pathogen being introduced from captivity into the wild, and calls for according biosecurity measures. The detection of *Bsal*-positive alive specimens of the hyper-susceptible fire salamander could indicate the existence of a less aggressive *Bsal* variant or the importance of environmental conditions for infection progression.

## Introduction

Modern amphibians (Lissamphibia), the oldest class of extant tetrapods^1^, have inhabited Earth since the Triassic and have survived several of the major mass extinction events^2^. However, they are presently one of the animal groups most threatened with extinction^3,4^. In 2017, the Global Amphibian Assessment revealed that 41% of the 6,609 species reviewed, showed evidence of decline, and 35 were already extinct^5^. While habitat degradation and destruction still appear to be the main culprits^6^, an emerging infectious disease, chytridiomycosis, is currently considered another major factor^7^, having led to numerous declines and extinctions worldwide^8–11^. Chytridiomycosis is the manifestation of an infection with one of two pathogens: *Batrachochytrium dendrobatidis* (*Bd*;^12,13^), a fungus that lethally infects many frogs (Anura)^12,14,15^ but also salamanders (Caudata)^16^ and caecilians (Gymnophiona)^17^; and *B. salamandrivorans* (*Bsal*;^10^) that appears to lethally affect only salamanders^18,19^. The clinical symptoms caused by these pathogens involve excessive skin shedding, and reddened or discoloured skin which, in *Bd*, leads to erythema and malfunctions in respiration and osmoregulation^12,20,21^; and, in *Bsal*, to deep epidermal ulcerations which are subsequently colonized by other microorganisms^10^. Several studies have explored methods to clear chytrid infections, with heat treatments and antifungals among the viable options for both pathogens^22,23^.

The chytrid fungus *Bd* has been detected in several parts of Europe^24–26^, inducing population declines in some cases but not leading to massive declines in the majority of species (with the exception of *Alytes* midwife toads in Spain^27^). *Bsal* was first detected as the cause of a mass-mortality event in fire salamanders (*Salamandra salamandra*) in the Netherlands^10,28^, but has now been detected also in Belgium and western Germany, causing mass mortality in fire salamanders but also infecting various newt species^29^. Currently, the potential spread of *Bsal*^30^ is one of the most pressing conservation challenges not only for European, but also for the conservation of the global salamander diversity^19^.

The import of newt and salamander species from Asia as pets, for research, or for zoological collections, is the hypothesized route of introduction of *Bsal* into European wildlife^18,31^. Therefore, captive collections of amphibians have the potential to be particularly relevant and potentially dangerous pathways for the further spread of *Bsal* into the wild. Currently, *Bsal* has been detected in captive collections in the United Kingdom and in Germany^32,33^, but not in the United States^34^.

After *Bsal* was demonstrated to occur in captive salamanders^32^, we carried out an additional study to assess the extent of occurrence of this pathogen in captive amphibian collections mainly within Germany. Here, we present the results of this study, based on 918 skin swabs taken from individual amphibians from captive collections screened for *Bd* and *Bsal* using quantitative PCR (qPCR), with numerous *Bsal* positives confirmed by direct sequencing and/or independent analysis of duplicate swabs in a second laboratory.

## Methods

Our study is based on samples taken by the private collection owners for routine health screenings. The owners of the collections check the individuals regularly for disease signs, such as skin ulcerations, lethargy, abnormal skin shedding and death. With the exception of the collection that experienced mass dying to a *Bsal* infection (Collection 1) at an earlier time point^32^, none of the clinical signs were observed in any other collection. In order to maintain the anonymity of collection owners, precise locations and species composition of the collections are not provided.

A total of 918 samples belonging to 111 species (11 Anura and 100 Caudata) and 46 subspecies (all Caudata) from 20 private captive amphibian collections were sampled between May of 2015 and May of 2016 (Supplementary Table 1 and Table 1). Eighteen of the collections were located in Germany (across nine federal states) and two in Sweden. All collection owners are members of the AG Urodela (‘Arbeitsgemeinschaft’ der Deutschen Gesellschaft für Herpetologie und Terrarienkunde e.V.), a working group of the German Society of Herpetology and Herpetoculture (DGHT). Members of the DGHT receive formation and guidelines on several aspects related to amphibians, and more specifically salamanders, including housing and how to carry out health checks. Housing conditions of amphibians varied among collections, with most terrestrial species being kept in groups of three or four individuals within glass terraria, while aquatic species were kept at higher densities, up to approximately 30 individuals. Enclosures ranged from 20 × 25 × 40 cm to 40 × 40 × 80 cm. Terrestrial and aquatic enclosures were equipped with access to hiding places.

**Table 1:** Prevalence of the chytrid fungi at each private collection. **N** indicates number of samples collected; **Pos**. refers to positives confirmed in both laboratories; and **Unc. Pos**. refers to unconfirmed positives. Collections 1, 3 and 4 were sampled a second time after a heat treatment was applied. (*) indicates collection on which mortality was detected^32^. Loads are presented as medians of zoospores per swab.

	**Origin**	**N**	Bd	**Load (IQR)**	Bsal	**Load (IQR)**	**Unc. Pos**.	**Load (IQR)**
			**Pos**.		**Pos**.			
**Collection1**	Hesse							
*May.2015		22	0	—	17	923.4 (1430.3)	4	1701.9 (2822.5)
Nov.2015		30	0	—	0	—	0	—
**Collection2**	North Rhine-Westphalia							
Jun.2015		3	0	—	0	—	0	—
**Collection3**	North Rhine-Westphalia							
Aug.2015		31	0	—	1	1.98	1	3.02
Oct.2015		16	0	—	0	—	0	—
**Collection4**	Lower Saxony							
Aug.2015		81	0	—	0	—	0	—
Nov.2015		13	0	—	0	—	0	—
**Collection5**	Saxony							
Aug.2015		105	0	—	1	2.6	3	0.1 (68.1)
Sep.2015		42	0	—	0	—	0	—
**Collection6**	Baden-Württemberg							
Aug.2015		26	0	—	0	—	0	—
**Collection7**	Saxony-Anhalt							
Aug.2015		32	2	10.1 (13.6)	0	—	0	—
**Collection8**	Saxony							
Aug.2015		25	0	—	2	1.6 (1.7)	1	0.1
**Collection9**	Baden-Württemberg							
Sep.2015		18	0	—	0	—	0	—
**Collection10**	Baden-Württemberg							
Sep.2015		30	3	10.1 (7.0)	0	—	0	—
**Collection11**	Baden-Württemberg							
Sep.2015		60	0	—	0	—	0	—
**Collection12**	Hesse							
Sep.2015		19	1	11.5	0	—	0	—
**Collection13**	North Rhine-Westphalia							
Oct.2015		7	0	—	1	203.8	0	—
**Collection14**	Rhineland-Palatinate							
Oct.2015		35	1	2.21	0	—	1	180.7
**Collection15**	Baden-Württemberg							
Oct.2015		25	0	—	0	—	0	—
**Collection16**	Bavaria							
Nov.2015		19	0	—	0	—	0	—
**Collection17**	Lower Saxony							
Nov.2015		61	0	—	1	2.4	5	4.6 (4.7)
**Collection18**	Baden-Württemberg							
Jan.2016		18	0	—	0	—	0	—
**Collection19**	Sweden							
05.2016		54	3	89.9 (334.7)	0	—	1	31.7
**Collection20**	Sweden							
May.2016		146	3	4.5 (15.5)	0	—	2	335.4 (344.3)
**TOTAL**		918	13	10.1 (19.2)	23	211.2 (1094.4)	18	5.7 (283.3)

From all captive collections, two individuals from every enclosure were sampled. Each individual was handled with clean nitrile gloves and its ventral surface was rubbed 10 times, simultaneously with two sterile rayon swabs (MW113; Medical Wire & Equipment, Corsham, UK). Each of these two swabs of an individual was kept separately in a sterile 1.5 ml centrifuge tube and stored at −20 °C until DNA extraction. A specific protocol for sampling that has been followed by breeders can be found in the supplementary materials. Additionally, one breeder (Collection 2) provided a clean swab as a sampling control, unfortunately other breeders failed to do so. The negative control was collected half way through the sampling event by opening a clean swab and placing it in a centrifuge tube. Three collections – also including Collection 1 - in which *Bsal* was detected were heat treated following established methods^23,35^. Individuals from this collection were re-sampled after the heat treatment following the same protocol as described above.

One swab per sampled individual was analysed in the laboratory of the Technical University Braunschweig (Braunschweig, Germany). Genomic DNA was extracted from the swabs using the Qiagen DNeasy Blood and Tissue Kit (Qiagen, Hilden, Germany) following the manufacturer’s Animal Tissues protocol with Pretreatment for Gram-Positive Bacteria. Incubation for the initial enzymatic lysis was extended to 1 h, and the temperature of the proteinase K lysis was increased to 70 °C to increase DNA yield^36^. One sterile swab was included in every third round of extraction as a negative control, with approximately 50 rounds being run.

For the samples that were found to be positive, DNA from the duplicate swab was extracted and analyzed using the same method but by a different person in the laboratory of Trier University (Trier, Germany), a laboratory accredited for the European *Bsal* initiative^37^. For nine samples for which no duplicate swab was available, such a double check was not possible. As an additional control, nine negatives were also analyzed in Trier, all of which were negative.

In both laboratories, qPCRs amplified a region of the ITS rRNA (120 bp), following a standard protocol^38^ with one alteration: the use of KlearKall Master Mix (LGC genomics, Middlesex, UK). Quantitative PCRs were performed on a CFX96 Real-Time System (Bio-Rad Laboratories Inc., Hercules, CA) in Braunschweig, and on a StepOnePlus (Applied Biosystems, Foster City, CA) in Trier. Each sample was run in duplicate in Braunschweig; when replicates of a sample showed contradictory results, a third replicate was run. Samples analyzed at Trier University were each run in triplicate. Each qPCR plate in Braunschweig had two replicates of *Bd* and *Bsal* standards (0.1–1,000 zoospores) and two negative controls, while in Trier each plate had *Bsal* standards in triplicate (0.1–1,000 zoospores) and three negative controls. Quantitative PCR amplification signals were only considered as positives when the signal was between the highest (1,000 zoospores) and the lowest standard (0.1 zoospores) and when the amplification curve was logarithmic. The estimated zoospore equivalents were converted to number of zoospores per swab (hereafter z/s) based on the extraction volume used. Loads were not normally distributed, so median and 75% inter quartile range (IQR) are reported.

Because our sampling approach involves two different swabs being taken from the same individual, one to be analysed initially in a laboratory, and the second one being analysed in a different laboratory to confirm the observations when the first one is positive, it is particularly robust against contamination during DNA extraction and subsequent manipulation of DNA templates (see Discussion). However, due to the lack of negative controls from the majority of breeders we cannot exclude contamination during sampling process. It must be emphasized that our approach also implies that differences among the results (but not confirmed by qPCR replication in a second laboratory) in the two laboratories can be expected, as with low infection loads, the amount of pathogen DNA might differ among the swabs. We considered samples as positive for *Bsal*, if they were positive in both laboratories; *unconfirmed positive* when they were only positive in one laboratory, and *negative* when they were negative in the first laboratory (only nine negative samples were repeated in the second laboratory, all of which were negative). For *Bd*, samples found positive in the first laboratory were not further tested, as our focus was on *Bsal*; thus, all *Bd* results are reported as *unconfirmed positives*.

From all *Bsal-*positive samples, stretches of DNA were sequenced to confirm its genetic identity with the *Bsal* type strain (Supplementary Table 3). Two regions were amplified, the ITS (Short: forward: 5′–TGC TCC ATC TCC CCC TCT TCA–3′, and reverse: 5′–TGA ACG CAC ATT GCA CTC TAC–3′; Long: forward: 5′–CAA CGG ATC TCT TGG CTC TC–3′, and reverse: 5′–GGT TTG CCT TAA TTT CAT AAT GG–3′) and the 28 S (forward: 5′-ACG CTT GAA ACC AGT ATT GAG TG–3′, and reverse: 5′–TAC AGC TGC GTT CCT CAG TC–3′) regions. The ITS short^38^ and the 28 S^39^ primers were as described in literature, while the ITS long was designed by us. Samples were enzymatically cleaned with ExoSap and sequenced using standard approaches on an ABI 3730xl capillary sequencer. Additionally negative controls were included to control for sequencing contamination. Sequences were controlled for quality by checking the chromatograms and were manually corrected when necessary in CodonCode Aligner. Subsequently sequences were blasted in the National Center for Biotechnology Information platform (https://blast.ncbi.nlm.nih.gov/Blast.cgi). The majority of samples yielded PCR products and could be successfully sequenced for at least one of these gene segments.

## Results

Of the 918 samples collected, 13 (1.4%) were unconfirmed positive for *Bd*, 23 (2.5%) were positive for *Bsal*, and 18 (2.0%) were unconfirmed positive for *Bsal*, with no co-occurrence of both pathogens in the same individual. Out of 28 of the positives (68%), 16 remained positive (39.0%) and 12 as unconfirmed positive (29.3%) after double checked at the Trier University. For all of these samples, DNA sequences matching *Bsal* were obtained (see below). The sampling control (a clean swab provided by breeder of Collection 2), the extraction controls, the qPCR controls and the sequencing controls were all negative.

None of the collections showed mortality or clinical signs of *Bd* or *Bsal* infection, including Collection 1 that had a *Bsal*-induced mortality event six months before for its *Salamandra* species^32^.

**Figure 1:**
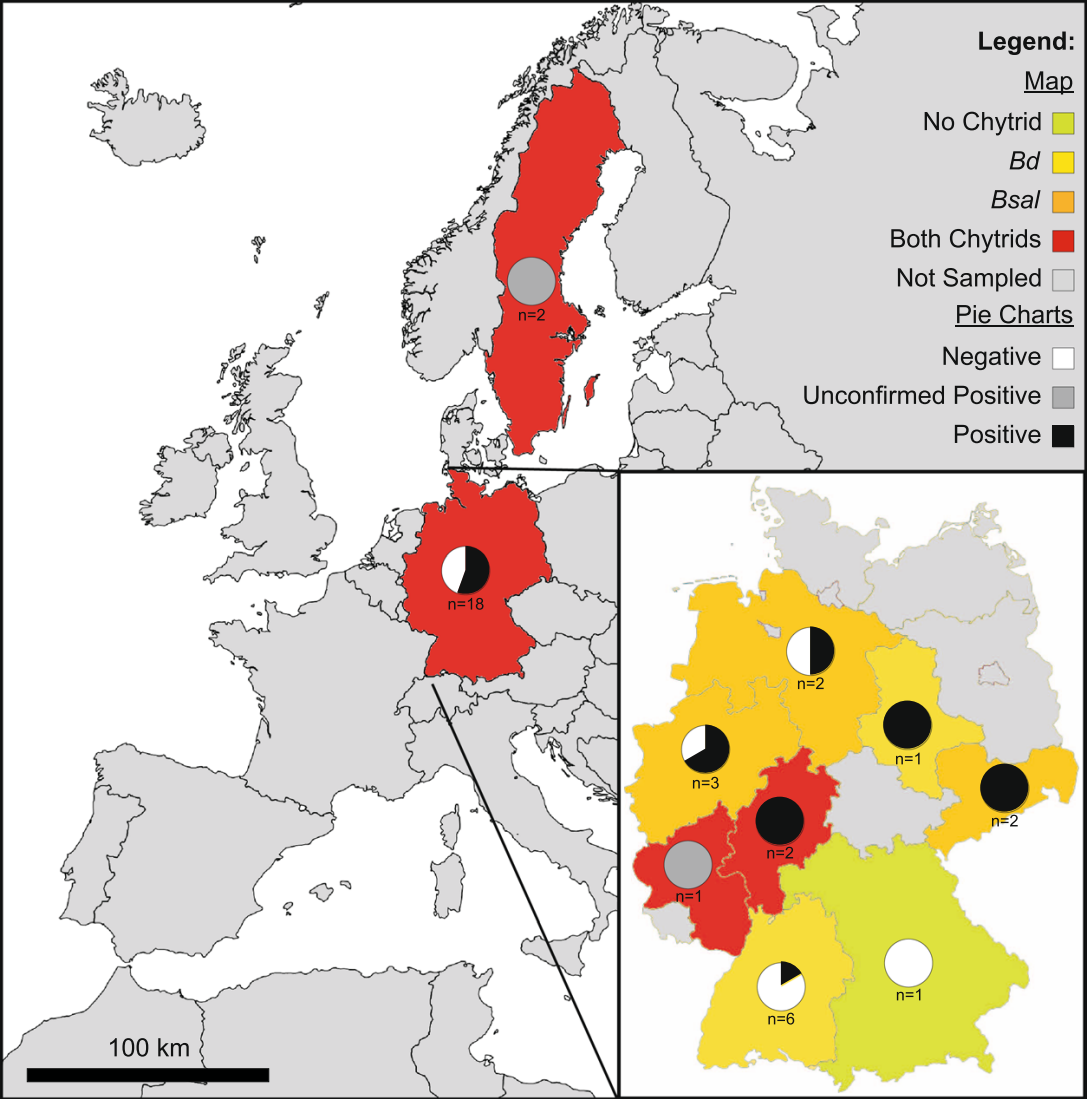
Distribution of positives and unconfirmed positive samples for both chytrid fungi, *Batrachochytrium dendrobatidis* (*Bd*) and *B. salamandrivorans* (*Bsal*), in captive collections from Germany and Sweden. Europe map (“leere Grundkarte von Europa”) was created by Ktrinko, is under Creative Commons Attribution-Share Alike 3.0 Unported license (https://creativecommons.org/licenses/by-sa/3.0/deed.en), can be found on https://commons.wikimedia.org/wiki/File:EuropaleereKarte.svg. Germany map (“Map of States of Germany (Area States & City States)”) was created by Roman Poulvas and David Liuzzo, is under Creative Commons Attribution-Share Alike 2.0 Germany license (https://creativecommons.org/licenses/by-sa/2.0/de/deed.en), can be found on https://en.wikipedia.org/wiki/File:Map_of_Lands_of_Germany_(Area_States_and_City_States).svg. Both maps were re-colored and elements (such as pie charts, legend and scale) added.

Both pathogens (*Bd* and *Bsal*) were detected in Germany (Fig. 1). The *Bsal* positives in Sweden could not be confirmed since no duplicate swab was available. Out of 20 collections sampled, six (30%) showed unconfirmed positive signals for *Bd* and nine (45%) showed positive and unconfirmed positive signals for *Bsal* (positive - 6 (30%); unconfirmed positive - 3 (15%)), with three private collections showing unconfirmed positive qPCR signals for both *Bd* and *Bsal* (Table 1). The median (IQR) loads (excluding the zeros of samples with three replicates) for *Bd* and *Bsal* were 101.1 (19.2) and 105.0 (965.8) (positive - 211.2 (1094.4); unconfirmed positive - 5.7 (283.3)) z/s, respectively.

Out of the 157 species and subspecies sampled, 11 (7.0%, three Anura and eight Caudata) showed unconfirmed positive signals for *Bd*, and 20 (12.7%, all Caudata) showed positive and unconfirmed positive signals for *Bsal* (positive − 12 (7.6%); unconfirmed positive – 8 (5.1%)) (Table 2). *Batrachochytrium dendrobatidis* was found (but not confirmed by qPCR replication in a second laboratory) in the following genera: *Alytes*, *Bombina* and *Gastrotheca* (Anura), and *Ichthyosaura*, *Neurergus*, *Salamandra*, *Salamandrella*, *Siren*, *Taricha* and *Triturus* (Caudata). Salamanders with *Bsal*-positives or unconfirmed positives belonged to the genera: *Ambystoma* (Ambytomatidae), *Cynops*, *Laotriton*, *Paramesotriton*, *Pleurodeles* and *Salamandra* (Salamandridae) (*Laotriton*, *Paramesotriton*, *Pleurodeles* only with unconfirmed positives). With the exception of the samples from the *Bsal* outbreak in Collection 1, the loads were below 400 z/s for both pathogens across the sampled species (below 200 when excluding the unconfirmed positives) (Supplementary Table 1).

**Table 2:** List of species positive (**Pos.**) or unconfirmed positive (**Unc. Pos.**) for the chytrid fungi, with number of examined individuals (**N**, positive/total), and median loads and inter quartile range (**Load (IQR)**, zoospores per swab) for *Batrachochytrium dendrobatidis* and *B. salamandrivorans* from private captive collections in Germany and Sweden. For full data see Supplementary Table 1.

	Species	Bd		Bsal			
		N	Load (IQR)	Pos.	Load (IQR)	Unc. Pos	Load (IQR)
Anura							
	*Alytes obstetricans*	1/1	22.4	0/1	0	0/1	0
	*Bombina bombina*	2/2	9.3 (0.8)	0/2	0	0/2	0
	*Gastrotheca riobambae*	2/5	380.6 (290.7)	0/5	0	0/5	0
Caudata							
	*Ambystoma maculatum*	0/4	0	1/4	98.5	0/4	0
	*Ambystoma opacum*	0/5	0	1/5	1.5	0/5	0
	*Cynops cyanurus*	0/5	0	1/5	3.2	0/5	0
	*Cynops e. ensicauda*	0/13	0	1/13	105	0/13	0
	*Ichthyosaura a. apuana*	1/5	11.5	0/5	0	0/5	0
	*Laotriton laoensis*	0/11	0	0/11	0	2/11	0.1 (0.1)
	*Neurergus kaiseri*	1/7	2.2	0/7	0	0/7	0
	*Paramesotriton deloustali*	0/7	0	0/7	0	1/7	3
	*Paramesotriton fuzhongensis*	0/4	0	0/4	0	1/4	4.7
	*Paramesotriton hongkongensis*	0/1	0	0/1	0	1/1	317.5
	*Pleurodeles nebulosus*	0/4	0	0/4	0	1/4	353.2
	*Pleurodeles waltl*	0/17	0	0/17	0	3/17	6.78 (759.8)
	*Salamandra a. pasubiensis*	0/5	0	0/5	0	1/5	31.7
	*Salamandra corsica*	1/15	32.4	0/15	0	0/15	0
	*Salamandra i. semenovi*	0/17	0	1/17	2.6	0/17	0
	*Salamandra s. almanzoris*	0/12	0	3/12	1430.0 (2171.0)	0/12	0
	*Salamandra s. bernardezi*	0/20	0	2/20	9.2 (12.8)	2/20	945.6 (1418.3)
	*Salamandra s. fastuosa*	0/17	0	2/17	945.9 (957.1)	0/17	0
	*Salamandra s. gallaica*	0/45	0	3/45	974.5 (2201.5)	2/45	2.0 (2.0)
	*Salamandra s. gigliolii*	0/30	0	2/30	8.0 (11.2)	0/30	0
	*Salamandra s. salamandra*	0/49	0	4/49	837.1 (1604.0)	2/49	2898.9 (4258.0)
	*Salamandra s. terrestris*	0/147	0	0/147	0	2/147	68.1 (102.1)
	*Salamandra s. werneri*	0/7	0	2/7	882.1 (1221.2)	0/7	0
	*Salamandrella keyserlingii*	1/3	4.5	0/3	0	0/3	0
	*Siren intermedia*	1/2	17.1	0/2	0	0/2	0
	*Taricha granulosa*	1/6	3.2	0/6	0	0/6	0
	*Triturus carnifex*	1/13	1.9	0/13	0	0/13	0

From all the *Bsal* positives and unconfirmed positives, 15 (65.2%) and 10 (55.5%) yielded sequences of good quality for the ITS region; and 14 (60.9%) and 4 (22.2%) for the 28 S region, respectively. All but three ITS sequences obtained fully matched the homologous sequence of the *Bsal* type strain (Genbank accession number KC762295) with 100% similarity, and all 28 S sequences fully matched the *Bsal* type strain sequence. Of the three deviant ITS sequences, one (from a skin swab of *A. maculatum*) had three nucleotide substitutions (positions 281, 283 and 314), one (from a skin swab of *Pleurodeles nebulosus*) had three heterozygous positions (positions 234, 235 and 312), and one (from a skin swab of *S. salamandra*) had one heterozygous position (position 383), compared to the *Bsal* type strain (Supplementary Table 3).

To three private collections (Collections 1, 3 and 5) on which *Bsal* was detected, a heat treatment was applied. These collections were sampled a second time afterwards. Collection 1 previously had high loads of *Bsal* (~1,000 zoospores) and suffered a mass mortality event (Table 1^32^), and Collections 3 and 5 had lower loads ( < 100 z/s), but individuals showed no clinical signs. All samples from the second sampling round of these three collections were negative for *Bsal* (Table 1). No further mortality events or clinical signs of *Bsal* infection were detected in these or any of the other sampled collections in the following ca. 18–24 months after sampling took place.

## Discussion

The chytrid fungus *Bd* is known to be widespread both in the wild and in captivity^15,40–42^. For *Bsal*, its distribution in the wild has been explored recently^29^, but its presence in captivity is restricted to only to two reports so far^32,33^. Our study indicates that *Bsal* might be more widespread in captivity than expected, although it appears to be absent in the USA^34^.

At present, and with the exception of Collection 1 at an earlier time point^32^, no clinical signs of chytridiomycosis have been reported from the captive collections studied herein, yet *Bsal* was detected in approximately one third of them, and approximately half, if also considering unconfirmed positives (Table 1). While interpretation of zoospore loads is difficult due to the semi-quantitative nature of qPCR and the potential for copy number variation^43,44^, it is worth noting that despite half of the loads being below 100 zoospores (lowest zoospore quantity known to cause mortality in amphibians^19^), some of the *Bsal*-positives were above that threshold in species such as the common fire salamander (*S. salamandra*) that are known to succumb to chytridiomycosis with lower loads^19^. One explanation for this lack of clinical signs is that some of the positives might be from a novel, less virulent strain of *Bsal*, or a different species of chytrid fungus that co-amplifies with the applied *Bsal* probe. This explanation might apply to some samples as the sequences obtained from the *A. maculatum*, *Pleurodeles nebulosus* and *S. salamandra Bsal*-positive samples yielded sequences slightly differing from the ITS rRNA sequence of the type strain (Supplementary Table 3). The possible existence of such genetically deviant strains requires further confirmation because due to insufficient DNA template it was not possible to repeat the respective PCRs, and accordingly artefacts through polymerase synthesis and/or subsequent sequencing errors thus cannot be fully excluded at this point. With that said, the remaining sequences obtained from *Bsal-*positive individuals yielded sequences 100% identical to the type strain of *Bsal* (Supplementary Table 3). The presence of asymptomatic positives in species that are susceptible to *Bsal*, such as *Salamandra* and *Pleurodeles*^18^, might also indicate that under certain conditions in captivity, these susceptible salamanders are able to tolerate and exist with the pathogen. This may be explained by adverse environmental conditions (such as higher temperatures and less water pools) that are not optimal for the pathogen, potentially making it difficult for *Bsal* to develop and disseminate rapidly and minimizing the escalation of the infection to clinical disease.

From the unconfirmed positives identified in this screening, 12 showed contradictory results (Supplementary Table 2). Two of these, amplified in the laboratory of the University of Trier had an amplification signal above the highest standard as in the laboratory of the Technical University of Braunschweig. It is expected that with an appropriate dilution these samples would have tested also positive in Braunschweig as well. Eight of the unconfirmed positives (five amplified in Trier, and three amplified in Braunschweig) presented loads below 7 zoospores. This variation in low-load positives is likely related to the standards varying slightly in the actual DNA present in them, and so samples that did not make the cut off in one laboratory, did so in the other. To minimize such issues, negative sampling controls are necessary, i.e. swabs taken out of their sterile envelope and placed into a vial, by the same person and at the same time the sampling is performed. Unfortunately, in this study the standardized sampling procedure as suggested by the DGHT missed this point. We would therefore like to point out that for future studies such negative controls should be taken. Given that gloves were changed between individuals and all involved breeders guaranteed to have followed the instructions, we believe that the possibility of cross contamination between individuals does not impact our main findings, i.e. our conclusion of *Bsal* being present in the respective collections.

We hypothesize these widespread qPCR positives indicate asymptomatic *Bsal* infections, but alternative interpretations require discussion. Although we cannot exclude sample cross-contamination during sampling, this factor should not affect our main conclusions as such contamination would only alter the species identification of the positive individuals, not the presence of *Bsal* in the respective collection. The possibility that our results are exclusively explained by false positives is unlikely for several reasons. First, none of the negative controls showed amplification for *Bsal* or *Bd*. Second, most replicated swabs showed the same results in both laboratories. Third, sequences from both regions (ITS and 28 S) of the positive and unconfirmed positive samples sequenced (positive – ITS: 15, 28 S: 14; and unconfirmed positives – ITS: 10, 28S: 4) matched to the *Bsal* type strain. Both regions were amplified as to control for the potential of false positives^39^. And finally, samples were processed simultaneously with approximately 1,000 other samples from the wild (partly included in Spitzen-van der Sluijs *et al*. and Sabino-Pinto *et al*.^29,45^), and none of these was found to be *Bsal*-positive, with the exception of 26 samples from one geographical region^29^ of a recent *Bsal* spread, also confirmed by the laboratory in Trier. Additionally, as discussed above, our results might reflect detection of less virulent strains of *Bsal* than those known to cause mass mortality in fire salamanders. It is important to emphasize again that our approach, taking two swabs simultaneously from the same individual, and separately analysing those in two different laboratories (including separate DNA extractions) differs from most qPCR replicate approaches in which merely the qPCR is repeated in a second laboratory often from the same extracted individual DNA template. Hence, our duplicate swabbing approach is particularly robust against contamination as it does not only exclude qPCR artefacts and contamination of reagents, but also contamination of the DNA template with *Bsal* DNA during DNA extraction and subsequent manipulation in the laboratory. Still, we also highlight once more that the lack of sampling controls, with the exception of the one provided by the breeder of Collection 2, does not completely exclude the potential of within-collection contamination.

In 2015, a private collection (Collection 1) showed high mortality as a result of *Bsal* infection^32^. Heat treatment is known to clear the fungus in laboratorial set-ups^23,35^, but their efficiency when applied under less strict conditions was until now not known. The private keeper of this collection increased the temperature of the rooms in which the salamanders were kept to ~25 °C for 10 days as suggested by Blooi *et al*.^35^. During the first five days of the treatment mortality decreased and stopped completely after that point. The same collection was re-sampled six months after the treatment after temperature had been reset to original conditions. Our results show that no sign of *Bsal* (or *Bd*) was detected in any of the sampled individuals, and no additional mortality in this collection has been reported to date. The same heat treatment was applied also to two other collections (Collections 3 and 5) in which asymptomatic *Bsal* infections were detected. Again, no positives were detected after the heat treatment (Table 1), confirming the effectiveness of this mitigation method in captivity.

Overall, approximately half of the collections sampled in this project were positive or unconfirmed positive for *Bd* and *Bsal*, with three collections having both pathogens (although not in the same individuals) (Table 1). This is a crucial information for the development of further mitigation strategies^46^. Raising awareness to the necessity of screening and quarantine is of paramount importance, as is the implementation of biosecurity measures. The apparent occurrence of asymptomatic infections poses a new challenge as infections might go undetected and thereby increase the likelihood of further transmission and spread of *Bsal* both in captivity (from exchanged salamanders and newts between collections) and into the wild.

The introduction of *Bsal* and possibly of other, discovered or yet undiscovered, pathogens via animal imports or existing captive collections into the wild can have devastating effects to native amphibian populations^10,18,28,29^. Additionally, its spread within collections can lead to mass mortality events^32^. We argue that *Bsal* should be declared and treated as an epizootic disease by the European Union and national countries, and recommend that quarantine and biosafety rules should be adapted and developed to limit the spread of *Bsal* through Europe. As a first step, the unregulated import of salamanders and newts potentially carrying *Bsal* into European countries should be stopped immediately – as it has been done in the case of the USA, Hungary and Switzerland already – until proper measures and safety rules can be applied.

### Availability of data and material

All data generated or analysed during this study are included in this published article and its supplementary information files.

## Electronic supplementary material

Supplementary Materials
