# Supplementary material for: Asymptomatic infection of the fungal pathogen Batrachochytrium salamandrivorans in captivity
Source: Sci Rep. 2018 Aug 6;8:11767. doi: 10.1038/s41598-018-30240-z (PMC6078946; doi:10.1038/s41598-018-30240-z)
Supplement: Supplementary file 1 — Supplementary Materials [file 41598_2018_30240_MOESM1_ESM.pdf]

SUPPLEMENTARY MATERIAL

Asymptomatic infection of the fungal pathogen *Batrachochytrium salamandrivorans* in captivity.

Joana Sabino-Pinto[1,*], Michael Veith[2], Miguel Vences[1], Sebastian Steinfartz[1]

[1] Zoological Institute, Technische Universität Braunschweig, Braunschweig, Germany

[2] Department of Biogeography, Trier University, Trier, Germany

* Corresponding author - Name: Joana Sabino Pinto. E-mail: joanasabinopinto@gmail.com

Swabbing guidelines

General considerations

- Sampling requires two participants: **Person A** to hold the individual and **Person B** to swab.
- Gloves that touched one individual should not touch another.
- Gloves have to be changed between individuals. Gloves that touched one individual cannot touch another.
- Each individual is simultaneously sampled with two swabs.
- Annotate (excel file) properly the numbers of the eppendorfs (identifying the swabbing pairs), date, species (subspecies if known), gender, original location (if known) and owner.
- Samples should be kept cooled (frozen if possible) at all times.

Protocol

1. **Person A** holds individual by the head and the base of the tail with the ventral surface facing up. Pressure can be applied so the individual will remain still while sampling.
2. **Person B** removes 2 swabs from the packages, holds them together and swabs ventral surface 10 x, while slightly turning the swab to allow the mucus to cover the whole tip. Each time consists of two strikes, one up and one down (Fig. 1).
3. **Person A** releases individual.
4. **Person B** places each single swab in a separate eppendorf tube, breaks swab stick and closes the eppendorf tube properly (Fig. 2).

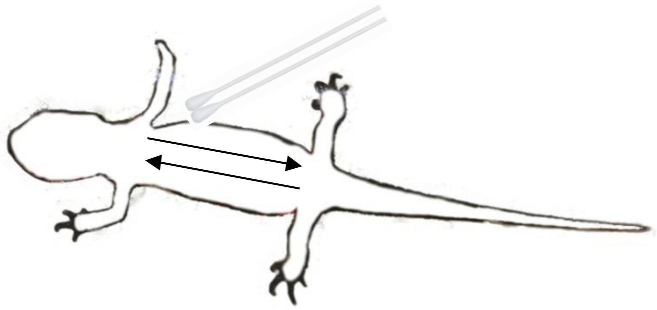

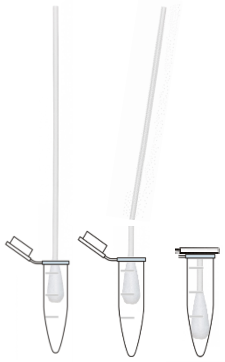

Supplementary Table 1. Species, number of individuals examined (positive/total) and median loads (zoospores per swab) for *Batrachochytrium dendrobatidis* and *B. salamandrivorans* from private captive collections from Germany and Sweden.

	Species	*Bd* N	Load (IQR)	*Bsal* Pos.	Load (IQR)	*Bsal* Unc. Pos.	Load (IQR)	Species	*Bd* N	Load (IQR)	*Bsal* Pos.	Load (IQR)	*Bsal* Unc. Pos.	Load (IQR)
Anura								*Amphiuma tridactylum*	0/2	0	0/2	0	0/2	0
	Alytes obstetricans	1/1	22.4	0/1	0	0/1	0	*Aneides lugubris*	0/5	0	0/5	0	0/5	0
	Barbarophryne brongersmai	0/3	0	0/3	0	0/3	0	*Aneides sp.*	0/2	0	0/2	0	0/2	0
	Bombina bombina	2/2	9.3 (0.8)	0/2	0	0/2	0	*Chioglossa lusitanica*	0/1	0	0/1	0	0/1	0
	Bombina orientalis	0/1	0	0/1	0	0/1	0	*Cynops cyanurus*	0/5	0	1/5	3.2	0/5	0
	Bombina variegata	0/10	0	0/10	0	0/10	0	*Cynops ensicauda*	0/8	0	0/8	0	0/8	0
	Bufo viridis	0/2	0	0/2	0	0/2	0	*Cynops e. ensicauda*	0/13	0	1/13	105	0/13	0
	Gastrotheca riobambae	2/5	380.6 (290.7)	0/5	0	0/5	0	*Cynops e. popei*	0/10	0	0/10	0	0/10	0
	Hyla arborea	0/2	0	0/2	0	0/2	0	*Cynops orientalis*	0/4	0	0/4	0	0/4	0
	Pelobates fuscus	0/2	0	0/2	0	0/2	0	*Cynops pyrrhogaster*	0/12	0	0/12	0	0/12	0
	Ranoidea aurea	0/3	0	0/3	0	0/3	0	*Cynops p. pyrrhogaster*	0/1	0	0/1	0	0/1	0
	Xenopus laevis	0/1	0	0/1	0	0/1	0	*Cynops p. sasayamae*	0/1	0	0/1	0	0/1	0
Caudata								*Desmognathus carolinensis*	0/2	0	0/2	0	0/2	0
	Ambystoma andersoni	0/2	0	0/2	0	0/2	0	*Dicamptodon tenebrosus*	0/4	0	0/4	0	0/4	0
	Ambystoma barbouri	0/1	0	0/1	0	0/1	0	*Euproctus platycephalus*	0/4	0	0/4	0	0/4	0
	Ambystoma californiense	0/2	0	0/2	0	0/2	0	*Eurycea quadridigitata*	0/1	0	0/1	0	0/1	0
	Ambystoma dumerilii	0/2	0	0/2	0	0/2	0	*Eurycea cirrigera*	0/1	0	0/1	0	0/1	0
	Ambystoma gracile	0/6	0	0/6	0	0/6	0	*Gyrinophilus porphyriticus*	0/2	0	0/2	0	0/2	0
	Ambystoma laterale	0/3	0	0/3	0	0/3	0	*Hemidactylium scutatum*	0/3	0	0/3	0	0/3	0
	Ambystoma macrodactylum	0/1	0	0/1	0	0/1	0	*Hynobius dunni*	0/1	0	0/1	0	0/1	0
	Ambystoma maculatum	0/4	0	1/4	98.5	0/4	0	*Hynobius quelpartensis*	0/1	0	0/1	0	0/1	0
	Ambystoma mavortium diaboli	0/1	0	0/1	0	0/1	0	*Hynobius cf. leechii*	0/1	0	0/1	0	0/1	0
	Ambystoma m. mavortium	0/9	0	0/9	0	0/9	0	*Hypselotriton cyanurus*	0/6	0	0/6	0	0/6	0
	Ambystoma m. melanostictum	0/1	0	0/1	0	0/1	0	*Hypselotriton c. yunnanensis*	0/6	0	0/6	0	0/6	0
	Ambystoma mexicanum	1/8	1.5	0/8	0	0/8	0	*Hypselotriton fudingensis*	0/2	0	0/2	0	0/2	0
	Ambystoma opacum	0/5	0	1/5	1.5	0/5	0	*Hypselotriton orientalis*	0/9	0	0/9	0	0/9	0
	Ambystoma tigrinum	0/2	0	0/2	0	0/2	0	*Ichthyosaura alpestris*	0/4	0	0/4	0	0/4	0
	Amphiuma means	0/1	0	0/1	0	0/1	0	*Ichthyosaura a. alpestris*	0/4	0	0/4	0	0/4	0

Supplementary Table 1. (cont.)

		Bd		*Bsal*		*Bsal*			*Bd*		*Bsal*		*Bsal*	
	**Species**	**N**	**Load (IQR)**	**N**	**Load (IQR)**	**N**	**Load (IQR)**	**Species**	**N**	**Load (IQR)**	**N**	**Load (IQR)**	**N**	**Load (IQR)**
Caudata								*Paradactylodon persicus*	0/2	0	0/2	0	0/2	0
	Ichthyosaura a. apuana	1/5	11.5	0/5	0	0/5	0	*Paramesotriton caudopunctatus*	0/1	0	0/1	0	0/1	0
	Ichthyosaura a. inexpectata	0/1	0	0/1	0	0/1	0	*Paramesotriton chinensis*	0/2	0	0/2	0	0/2	0
	Ichthyosaura a. montenegrina	0/2	0	0/2	0	0/2	0	*Paramesotriton deloustali*	0/7	0	0/7	0	1/7	3
	Ichthyosaura a. reiseri	0/2	0	0/2	0	0/2	0	*Paramesotriton fuzhongensis*	0/4	0	0/4	0	1/4	4.7
	Ichthyosaura a. veluchiensis	0/1	0	0/1	0	0/1	0	*Paramesotriton hongkongensis*	0/1	0	0/1	0	1/1	317.5
	Laotriton laoensis	0/11	0	0/11	0	2/11	0.1 (0.1)	*Paramesotriton labiatus*	0/5	0	0/5	0	0/5	0
	Liangshantriton taliangensis	0/1	0	0/1	0	0/1	0	*Paramesotriton qixilingensis*	0/1	0	0/1	0	0/1	0
	Lissotriton boscai	0/2	0	0/2	0	0/2	0	*Plethodon cinereus*	0/1	0	0/1	0	0/1	0
	Lissotriton helveticus	0/3	0	0/3	0	0/3	0	*Plethodon glutinosus*	0/2	0	0/2	0	0/2	0
	Lissotriton h. alonsoi	0/2	0	0/2	0	0/2	0	*Pleurodeles nebulosus*	0/4	0	0/4	0	1/4	353.2
	Lissotriton italicus	0/2	0	0/2	0	0/2	0	*Pleurodeles waltl*	0/17	0	0/17	0	3/17	6.78 (759.8)
	Lissotriton montandoni	0/2	0	0/2	0	0/2	0	*Pseudotriton ruber*	0/2	0	0/2	0	0/2	0
	Lissotriton vulgaris vulgaris	0/3	0	0/3	0	0/3	0	*Pseudotriton r. schencki*	0/2	0	0/2	0	0/2	0
	Lyciasalamandra fazilae	0/2	0	0/2	0	0/2	0	*Salamandra algira*	0/2	0	0/2	0	0/2	0
	Mertensiella caucasica	0/2	0	0/2	0	0/2	0	*Salamandra a. algira*	0/2	0	0/2	0	0/2	0
	Necturus maculosus	0/2	0	0/2	0	0/2	0	*Salamandra a. splendens*	0/1	0	0/1	0	0/1	0
	Neurergus crocatus	0/8	0	0/8	0	0/8	0	*Salamandra a. tingitana*	0/8	0	0/8	0	0/8	0
	Neurergus kaiseri	1/7	2.2	0/7	0	0/7	0	*Salamandra atra*	0/12	0	0/12	0	0/12	0
	Neurergus strauchii	0/5	0	0/5	0	0/5	0	*Salamandra a. atra*	0/6	0	0/6	0	0/6	0
	Notophtalmus viridescens	0/2	0	0/2	0	0/2	0	*Salamandra a. aurorae*	0/13	0	0/13	0	0/13	0
	Notophthalmus v. louisianensis	0/2	0	0/2	0	0/2	0	*Salamandra a. pasubiensis*	0/5	0	0/5	0	1/5	31.7
	Notophthalmus v. piaropicola	0/3	0	0/3	0	0/3	0	*Salamandra corsica*	1/15	32.4	0/15	0	0/15	0
	Notophthalmus v. viridescens	0/4	0	0/4	0	0/4	0	*Salamandra infraimmaculata*	0/3	0	0/3	0	0/3	0
	Ommatotriton ophryticus	0/1	0	0/1	0	0/1	0	*Salamandra i. infraimmaculata*	0/18	0	0/18	0	0/18	0
	Ommatotriton vittatus	0/1	0	0/1	0	0/1	0	*Salamandra i. orientalis*	0/4	0	0/4	0	0/4	0
	Ommatotriton sp.	0/2	0	0/2	0	0/2	0	*Salamandra i. semenovi*	0/17	0	1/17	2.6	0/17	0
	Onychodactylus fuscus	0/2	0	0/2	0	0/2	0	*Salamandra lanzai*	0/6	0	0/6	0	0/6	0

Supplementary Table 1. (cont.)

	Species	*Bd* N	*Bd* Load (IQR)	*Bsal* N	*Bsal* Load (IQR)	*Bsal* N	*Bsal* Load (IQR)	Species	*Bd* N	*Bd* Load (IQR)	*Bsal* N	*Bsal* Load (IQR)	*Bsal* N	*Bsal* Load (IQR)
Caudata								*Taricha torosa*	0/1	0	0/1	0	0/1	0
	Salamandra perspicillata	0/1	0	0/1	0	0/1	0	*Triturus carnifex*	1/13	1.9	0/13	0	0/13	0
	Salamandra salamandra	0/3	0	0/3	0	0/3	0	*Triturus cristatus*	0/9	0	0/9	0	0/9	0
	Salamandra s. alfredschmidti	0/7	0	0/7	0	0/7	0	*Triturus dobrogicus*	0/5	0	0/5	0	0/5	0
	Salamandra s. almanzoris	0/12	0	3/12	1430.0 (2171.0)	0/12	0	*Triturus d. macrosoma*	0/3	0	0/3	0	0/3	0
	Salamandra s. bejarae	0/7	0	0/7	0	0/7	0	*Triturus ivanbureschi*	0/2	0	0/2	0	0/2	0
	Salamandra s. bernardezi	0/20	0	2/20	9.2 (12.8)	2/20	945.6 (1418.3)	*Triturus karelinii*	0/6	0	0/6	0	0/6	0
	Salamandra s. beschkovi	0/8	0	0/8	0	0/8	0	*Triturus macedonicus*	0/2	0	0/2	0	0/2	0
	Salamandra s. corsica	0/2	0	0/2	0	0/2	0	*Triturus marmoratus*	0/9	0	0/9	0	0/9	0
	Salamandra s. crespoi	0/9	0	0/9	0	0/9	0	*Triturus pygmaeus*	0/3	0	0/3	0	0/3	0
	Salamandra s. fastuosa	0/17	0	2/17	945.9 (957.1)	0/17	0	*Triturus "candidate sp."*	0/1	0	0/1	0	0/1	0
	Salamandra s. gallaica	0/45	0	3/45	974.5 (2201.5)	2/45	2.0 (2.0)	*Triturus dobrogicus/carnifez*	0/2	0	0/2	0	0/2	0
	Salamandra s. gigliolii	0/30	0	2/30	8.0 (11.2)	0/30	0	*Triturus dobrogicus/carolinensis*	0/2	0	0/2	0	0/2	0
	Salamandra s. longirostris	0/4	0	0/4	0	0/4	0	*Triturus dobrogicus/cristatus*	0/2	0	0/2	0	0/2	0
	Salamandra s. morenica	0/13	0	0/13	0	0/13	0	*Tylototriton himalayensis/uyenoi*	0/2	0	0/2	0	0/2	0
	Salamandra s. salamandra	0/49	0	4/49	837.1 (1604.0)	2/49	2898.9 (4258.0)	*Tylototriton guangxiensis*	0/2	0	0/2	0	0/2	0
	Salamandra s. terrestris	0/147	0	0/147	0	2/147	68.1 (102.1)	*Tylototriton kweichowensis*	0/2	0	0/2	0	0/2	0
	Salamandra s. werneri	0/7	0	2/7	882.1 (1221.2)	0/7	0	*Tylototriton lizhenchangi*	0/1	0	0/1	0	0/1	0
	Salamandrella keyserlingii	1/3	4.5	0/3	0	0/3	0	*Tylototriton shanjing*	0/1	0	0/1	0	0/1	0
	Siren intermedia	1/2	17.1	0/2	0	0/2	0	*Tylototriton shanorum*	0/3	0	0/3	0	0/3	0
	Siren i. intermedia	0/1	0	0/1	0	0/1	0	*Tylototriton verrucosus*	0/7	0	0/7	0	0/7	0
	Siren lacertina	0/1	0	0/1	0	0/1	0	*Tylototriton yangi*	0/2	0	0/2	0	0/2	0
	Speleomantes italicus	0/2	0	0/2	0	0/2	0	*Tylototriton cf. shanjing/uyenoi*	0/1	0	0/1	0	0/1	0
	Speleomantes strinatii	0/2	0	0/2	0	0/2	0	*Typhlonectes natans*	0/1	0	0/1	0	0/1	0
	Taricha granulosa	1/6	3.2	0/6	0	0/6	0							

A/B Hybrid individuals between species A and B

Supplementary Table 2. Species of the positive samples and median loads (zoospores per swab) estimated in both laboratories.

**Species**	**Braunschweig**	**Trier**
Ambystoma maculatum	145.50	51.50
Ambystoma opacum	1.52	-
Cynops cyanurus	5.06	1.35
Cynops ensicauda ensicauda	196.12	13.97
Laotriton laoensis	0.00	0.10
Laotriton laoensis	0.00	0.10
Paramesotriton deloustali	3.02	-
Paramesotriton fuzhongensis	4.71	0.00
Paramesotriton hongkongensis	317.48	-
Pleurodeles nebulosus	353.22	-
Pleurodeles waltl	4.56	0.00
Pleurodeles waltl	6.78	0.00
Pleurodeles waltl	0.00	1512.83
Salamandra atra pasubiensis	31.70	-
Salamandra infraimmaculata semenovi	5.10	0.10
Salamandra s. almanzoris	3.73	1.00
Salamandra s. almanzoris	2358.74	501.80
Salamandra s. almanzoris	5112.26	711.20
Salamandra s. bernardezi	1.98	-
Salamandra s. bernardezi	29.83	3.04
Salamandra s. bernardezi	0.00	0.10
Salamandra s. bernardezi	0.00	1891.00
Salamandra s. fastuosa	1515.87	330.90
Salamandra s. fastuosa	1754.46	182.34
Salamandra s. gallaica	461.18	53.15
Salamandra s. gallaica	1706.37	242.60
Salamandra s. gallaica	5348.66	1508.48
Salamandra s. gallaica	0.00	2.00
Salamandra s. gallaica	2.04	-
Salamandra s. gigliolii	1.74	-
Salamandra s. gigliolii	19.00	9.59
Salamandra s. salamandra	202.02	220.34
Salamandra s. salamandra	798.04	121.79
Salamandra s. salamandra	2088.76	340.00
Salamandra s. salamandra	3566.97	1978.73
Salamandra s. salamandra	180.65	0.00
Salamandra s. salamandra	0.00	5617.06
Salamandra s. terrestris	0.00	0.10
Salamandra s. terrestris	136.11	-
Salamandra salamandra werneri	407.49	0.10
Salamandra salamandra werneri	2522.82	597.90

- Sample not analysed

Supplementary Table 3. Sequences of *Batrachochytrium salamandrivorans* positives obtained from swabs of captive salamanders and sequence identity to the type strain of *Bsal* (Genbank accession number NR_111867.1). The two sequences from *Salamandra salamandra* originate from the same individual but from independent samples. Nucleotides underlined in *Ambystoma maculatum* are mutations from the original *Bsal* sequence.

Sample	*5.8S Primer*	*5.8S Sequence*	*Blast %*	*28S Sequence*	*Blast %*
Salamandra salamandra	Long	AAAAAGACGAGAAAGATATTCATTTTTGAATCCATGATTTAGTTTGTCTACTATGAATAGATGCGAATAAAACCACATTCAATTACATTTCGACAAAAAAAGGGAATTAAAAGTTTTCTCCTTCAACTTGATTTAAAGCGAGTTGGTTTTCTTTTGAAAAAAAGAACCTCAACTAATCACTCATTATCTGCTCCATCTCCCCCTCTTCATCCCTAACCCTATTTTTATATCACTTTTTAGATGATATAAAAAGACAAGGAAATGAATTAAAAAAAGAAAAATAGAACAAGAAAATACTATTGATTCTCAAACAGGCATACTCTACAAAGTAGAGTGCAATGTGCGTTCAAAGATTCGATGATTCACTGAGTTCTGCAATTCACATTACGTATCGCATTTCG	100	TGCGTTCCTCAGTCCAGATCATGGCATAATGTCAATAAACTATAACACCCCAGAATAAATCCTGGGGCTACCTTTCTAAAGACTTTTTACCACGTTCCAAACTGACGCTGACTCGAGATAACTGAAAAGTGCATACCCCCCTTCTCTCTTGCGAAAGAAACGGAATACTGAATTTCATCACTCAATACTGGTTTCA	100
Salamandra salamandra	Long	AAAAAGACGAGAAAGATATTCATTTTTGAATCCATGATTTAGTTTGTCTACTATGAATAGATGCGAATAAAACCACATTCAATTACATTTCGACAAAAAAAGGGAATTAAAAGTTTTCTCCTTCAACTTGATTTAAAGCGAGTTGGTTTTCTTTTGAAAAAAAGAACCTCAACTAATCACTCATTATCTGCTCCATCTCCCCCTCTTCATCCCTAACCCTATTTTTATATCACTTTTTAGATGATATAAAAAGACAAGGAAATGAATTAAAAAAAGAAAAATAGAACAAGAAAATACTATTGATTCTCAAACAGGCATACTCTACAAAGTAGAGTGCAATGTGCGTTCAAAGATTCGATGATTCACTGAGTTCTGCAATTCACATTACGTATCGCATTTCG	100	TGCGTTCCTCAGTCCAGATCATGGCATAATGTCAATAAACTATAACACCCCAGAATAAATCCTGGGGCTACCTTTCTAAAGACTTTTTACCACGTTCCAAACTGACGCTGACTCGAGATAACTGAAAAGTGCATACCCCCCTTCTCTCTTGCGAAAGAAACGGAATACTGAATTTCATCACTCAATACTGGTTTCA	100
Salamandra salamandra	Short	AACCACATTCAATTACATTTCGACAAAAAAAGGGAATTAAAAGTTTTCTCCTTCAACTTGATTTAAAGCGAGTTGGTTTTCTTTTGAAAAAAAGAACCTCAACTAATCACTCATTATCTGCTCCATCTCCCCCTCTTCATCCCTAACCCTATTTTTATATCACTTTTTAGATGATATAAAAAGACAAGGAAATGAATTAAAAAAAGAAAAATAGAACAAGAAAATACTATTGATTCTCAAACAGGCATACTCTACAAAGTAGAGTGCAATGTGCGTTCAAAGATTCGATGATTCACTGAGTTCTGCAATTCA**M**ATTACGTATCGCATTTCG	99	TGCGTTCCTCAGTCCAGATCATGGCATAATGTCAATAAACTATAACACCCCAGAATAAATCCTGGGGCTACCTTTCTAAAGACTTTTTACCACGTTCCAAACTGACGCTGACTCGAGATAACTGAAAAGTGCATACCCCCCTTCTCTCTTGCGAAAGAAACGGAATACTGAATTTCATCACTCAATACTGGTTTCA	100
Salamandra salamandra				TGCGTTCCTCAGTCCAGATCATGGCATAATGTCAATAAACTATAACACCCCAGAATAAATCCTGGGGCTACCTTTCTAAAGACTTTTTACCACGTTCCAAACTGACGCTGACTCGAGATAACTGAAAAGTGCATACCCCCCTTCTCTCTTGCGAAAGAAACGGAATACTGAATTTCATCACTCAATACTGGTTTCA	100
Salamandra salamandra	Short	GCTCCATCTCCCCCTCTTCATCCCTAACCCTATTTTTATATCACTTTTTAGATGATATAAAAAGACAAGGAAATGAATTAAAAAAAGAAAAATAGAACAAGAAAATACTATTGATTCTCAAACAGGCATACTCTACAAAGTAGAGTGCAATGTGCGTTC	100	TGCGTTCCTCAGTCCAGATCATGGCATAATGTCAATAAACTATAACACCCCAGAATAAATCCTGGGGCTACCTTTCTAAAGACTTTTTACCACGTTCCAAACTGACGCTGACTCGAGATAACTGAAAAGTGCATACCCCCCTTCTCTCTTGCGAAAGAAACGGAATACTGAATTTCATCACTCAATACTGGTTTCA	100
Salamandra salamandra	Long	AAAAAGACGAGAAAGATATTCATTTTTGAATCCATGATTTAGTTTGTCTACTATGAATAGATGCGAATAAAACCACATTCAATTACATTTCGACAAAAAAAGGGAATTAAAAGTTTTCTCCTTCAACTTGATTTAAAGCGAGTTGGTTTTCTTTTGAAAAAAAGAACCTCAACTAATCACTCATTATCTGCTCCATCTCCCCCTCTTCATCCCTAACCCTATTTTTATATCACTTTTTAGATGATATAAAAAGACAAGGAAATGAATTAAAAAAAGAAAAATAGAACAAGAAAATACTATTGATTCTCAAACAGGCATACTCTACAAAGTAGAGTGCAATGTGCGTTCAAAGATTCGATGATTCACTGAGTTCTGCAATTCACATTACGTATCGCATTTCG	100	TGCGTTCCTCAGTCCAGATCATGGCATAATGTCAATAAACTATAACACCCCAGAATAAATCCTGGGGCTACCTTTCTAAAGACTTTTTACCACGTTCCAAACTGACGCTGACTCGAGATAACTGAAAAGTGCATACCCCCCTTCTCTCTTGCGAAAGAAACGGAATACTGAATTTCATCACTCAATACTGGTTTCA	100
Salamandra salamandra	Short	TCTTGAAATTTAAATCACCTTAGTTAAATGCATATAGGACTACTTGGCAAATGACTTAAAAACACAAGGTTACAACAAGAAAATACTATTGATTCTCAAACAGGCATACTCTACAAAGTAGAGTGCAATGTGCGTTCAAA	100	TGCGTTCCTCAGTCCAGATCATGGCATAATGTCAATAAACTATAACACCCCAGAATAAATCCTGGGGCTACCTTTCTAAAGACTTTTTACCACGTTCCAAACTGACGCTGACTCGAGATAACTGAAAAGTGCATACCCCCCTTCTCTCTTGCGAAAGAAACGGAATACTGAATTTCATCACTCAATACTGGTTTCA	100
Salamandra salamandra	Short	TCTTGAAATTTAAATCACCTTAGTTAAATGCATATAGGACTACTTGGCAAATGACTTAAAAACACAAGGTTACAACAAGAAAATACTATTGATTCTCAAACAGGCATACTCTACAAAGTAGAGTGCAATGTGCGTTCAAA	100	TGCGTTCCTCAGTCCAGATCATGGCATAATGTCAATAAACTATAACACCCCAGAATAAATCCTGGGGCTACCTTTCTAAAGACTTTTTACCACGTTCCAAACTGACGCTGACTCGAGATAACTGAAAAGTGCATACCCCCCTTCTCTCTTGCGAAAGAAACGGAATACTGAATTTCATCACTCAATACTGGTTTCA	100
Salamandra salamandra	Long	AAAAAGACGAGAAAGATATTCATTTTTGAATCCATGATTTAGTTTGTCTACTATGAATAGATGCGAATAAAACCACATTCAATTACATTTCGACAAAAAAAGGGAATTAAAAGTTTTCTCCTTCAACTTGATTTAAAGCGAGTTGGTTTTCTTTTGAAAAAAAGAACCTCAACTAATCACTCATTATCTGCTCCATCTCCCCCTCTTCATCCCTAACCCTATTTTTATATCACTTTTTAGATGATATAAAAAGACAAGGAAATGAATTAAAAAAAGAAAAATAGAACAAGAAAATACTATTGATTCTCAAACAGGCATACTCTACAAAGTAGAGTGCAATGTGCGTTCAAAGATTCGATGATTCACTGAGTTCTGCAATTCACATTACGTATCGCATTTCG	100	TGCGTTCCTCAGTCCAGATCATGGCATAATGTCAATAAACTATAACACCCCAGAATAAATCCTGGGGCTACCTTTCTAAAGACTTTTTACCACGTTCCAAACTGACGCTGACTCGAGATAACTGAAAAGTGCATACCCCCCTTCTCTCTTGCGAAAGAAACGGAATACTGAATTTCATCACTCAATACTGGTTTCA	100
Salamandra salamandra	Long	AAAAAGACGAGAAAGATATTCATTTTTGAATCCATGATTTAGTTTGTCTACTATGAATAGATGCGAATAAAACCACATTCAATTACATTTCGACAAAAAAAGGGAATTAAAAGTTTTCTCCTTCAACTTGATTTAAAGCGAGTTGGTTTTCTTTTGAAAAAAAGAACCTCAACTAATCACTCATTATCTGCTCCATCTCCCCCTCTTCATCCCTAACCCTATTTTTATATCACTTTTTAGATGATATAAAAAGACAAGGAAATGAATTAAAAAAAGAAAAATAGAACAAGAAAATACTATTGATTCTCAAACAGGCATACTCTACAAAGTAGAGTGCAATGTGCGTTCAAAGATTCGATGATTCACTGAGTTCTGCAATTCACATTACGTATCGCATTTCG	100	TGCGTTCCTCAGTCCAGATCATGGCATAATGTCAATAAACTATAACACCCCAGAATAAATCCTGGGGCTACCTTTCTAAAGACTTTTTACCACGTTCCAAACTGACGCTGACTCGAGATAACTGAAAAGTGCATACCCCCCTTCTCTCTTGCGAAAGAAACGGAATACTGAATTTCATCACTCAATACTGGTTTCA	100
Salamandra salamandra	Short	TCTTGAAATTTAAATCACCTTAGTTAAATGCATATAGGACTACTTGGCAAATGACTTAAAAACACAAGGTTACAACAAGAAAATACTATTGATTCTCAAACAGGCATACTCTACAAAGTAGAGTGCAATGTGCGTTCAAA	100		
Salamandra salamandra	Long	AAAAAGACGAGAAAGATATTCATTTTTGAATCCATGATTTAGTTTGTCTACTATGAATAGATGCGAATAAAACCACATTCAATTACATTTCGACAAAAAAAGGGAATTAAAAGTTTTCTCCTTCAACTTGATTTAAAGCGAGTTGGTTTTCTTTTGAAAAAAAGAACCTCAACTAATCACTCATTATCTGCTCCATCTCCCCCTCTTCATCCCTAACCCTATTTTTATATCACTTTTTAGATGATATAAAAAGACAAGGAAATGAATTAAAAAAAGAAAAATAGAACAAGAAAATACTATTGATTCTCAAACAGGCATACTCTACAAAGTAGAGTGCAATGTGCGTTCAAAGATTCGATGATTCACTGAGTTCTGCAATTCACATTACGTATCGCATTTCG	100		
Salamandra salamandra	Long	AAAAAGACGAGAAAGATATTCATTTTTGAATCCATGATTTAGTTTGTCTACTATGAATAGATGCGAATAAAACCACATTCAATTACATTTCGACAAAAAAAGGGAATTAAAAGTTTTCTCCTTCAACTTGATTTAAAGCGAGTTGGTTTTCTTTTGAAAAAAAGAACCTCAACTAATCACTCATTATCTGCTCCATCTCCCCCTCTTCATCCCTAACCCTATTTTTATATCACTTTTTAGATGATATAAAAAGACAAGGAAATGAATTAAAAAAAGAAAAATAGAACAAGAAAATACTATTGATTCTCAAACAGGCATACTCTACAAAGTAGAGTGCAATGTGCGTTCAAAGATTCGATGATTCACTGAGTTCTGCAATTCACATTACGTATCGCATTTCG	100	TGCGTTCCTCAGTCCAGATCATGGCATAATGTCAATAAACTATAACACCCCAGAATAAATCCTGGGGCTACCTTTCTAAAGACTTTTTACCACGTTCCAAACTGACGCTGACTCGAGATAACTGAAAAGTGCATACCCCCCTTCTCTCTTGCGAAAGAAACGGAATACTGAATTTCATCACTCAATACTGGTTTCA	100
Salamandra salamandra	Long	AAAAAGACGAGAAAGATATTCATTTTTGAATCCATGATTTAGTTTGTCTACTATGAATAGATGCGAATAAAACCACATTCAATTACATTTCGACAAAAAAAGGGAATTAAAAGTTTTCTCCTTCAACTTGATTTAAAGCGAGTTGGTTTTCTTTTGAAAAAAAGAACCTCAACTAATCACTCATTATCTGCTCCATCTCCCCCTCTTCATCCCTAACCCTATTTTTATATCACTTTTTAGATGATATAAAAAGACAAGGAAATGAATTAAAAAAAGAAAAATAGAACAAGAAAATACTATTGATTCTCAAACAGGCATACTCTACAAAGTAGAGTGCAATGTGCGTTCAAAGATTCGATGATTCACTGAGTTCTGCAATTCACATTACGTATCGCATTTCG	100		
Salamandra salamandra	Long	AAAAAGACGAGAAAGATATTCATTTTTGAATCCATGATTTAGTTTGTCTACTATGAATAGATGCGAATAAAACCACATTCAATTACATTTCGACAAAAAAAGGGAATTAAAAGTTTTCTCCTTCAACTTGATTTAAAGCGAGTTGGTTTTCTTTTGAAAAAAAGAACCTCAACTAATCACTCATTATCTGCTCCATCTCCCCCTCTTCATCCCTAACCCTATTTTTATATCACTTTTTAGATGATATAAAAAGACAAGGAAATGAATTAAAAAAAGAAAAATAGAACAAGAAAATACTATTGATTCTCAAACAGGCATACTCTACAAAGTAGAGTGCAATGTGCGTTCAAAGATTCGATGATTCACTGAGTTCTGCAATTCACATTACGTATCGCATTTCG	100	TGCGTTCCTCAGTCCAGATCATGGCATAATGTCAATAAACTATAACACCCCAGAATAAATCCTGGGGCTACCTTTCTAAAGACTTTTTACCACGTTCCAAACTGACGCTGACTCGAGATAACTGAAAAGTGCATACCCCCCTTCTCTCTTGCGAAAGAAACGGAATACTGAATTTCATCACTCAATACTGGTTTCA	100
Salamandra salamandra	Long	AAAAAGACGAGAAAGATATTCATTTTTGAATCCATGATTTAGTTTGTCTACTATGAATAGATGCGAATAAAACCACATTCAATTACATTTCGACAAAAAAAGGGAATTAAAAGTTTTCTCCTTCAACTTGATTTAAAGCGAGTTGGTTTTCTTTTGAAAAAAAGAACCTCAACTAATCACTCATTATCTGCTCCATCTCCCCCTCTTCATCCCTAACCCTATTTTTATATCACTTTTTAGATGATATAAAAAGACAAGGAAATGAATTAAAAAAAGAAAAATAGAACAAGAAAATACTATTGATTCTCAAACAGGCATACTCTACAAAGTAGAGTGCAATGTGCGTTCAAAGATTCGATGATTCACTGAGTTCTGCAATTCACATTACGTATCGCATTTCG	100	TGCGTTCCTCAGTCCAGATCATGGCATAATGTCAATAAACTATAACACCCCAGAATAAATCCTGGGGCTACCTTTCTAAAGACTTTTTACCACGTTCCAAACTGACGCTGACTCGAGATAACTGAAAAGTGCATACCCCCCTTCTCTCTTGCGAAAGAAACGGAATACTGAATTTCATCACTCAATACTGGTTTCA	100
Salamandra salamandra	Short	TCTTGAAATTTAAATCACCTTAGTTAAATGCATATAGGACTACTTGGCAAATGACTTAAAAACACAAGGTTACAACAAGAAAATACTATTGATTCTCAAACAGGCATACTCTACAAAGTAGAGTGCAATGTGCGTTCAAA	100	TGCGTTCCTCAGTCCAGATCATGGCATAATGTCAATAAACTATAACACCCCAGAATAAATCCTGGGGCTACCTTTCTAAAGACTTTTTACCACGTTCCAAACTGACGCTGACTCGAGATAACTGAAAAGTGCATACCCCCCTTCTCTCTTGCGAAAGAAACGGAATACTGAATTTCATCACTCAATACTGGTTTCA	100
Salamandra salamandra	Long	AAAAAGACGAGAAAGATATTCATTTTTGAATCCATGATTTAGTTTGTCTACTATGAATAGATGCGAATAAAACCACATTCAATTACATTTCGACAAAAAAAGGGAATTAAAAGTTTTCTCCTTCAACTTGATTTAAAGCGAGTTGGTTTTCTTTTGAAAAAAAGAACCTCAACTAATCACTCATTATCTGCTCCATCTCCCCCTCTTCATCCCTAACCCTATTTTTATATCACTTTTTAGATGATATAAAAAGACAAGGAAATGAATTAAAAAAAGAAAAATAGAACAAGAAAATACTATTGATTCTCAAACAGGCATACTCTACAAAGTAGAGTGCAATGTGCGTTCAAAGATTCGATGATTCACTGAGTTCTGCAATTCACATTACGTATCGCATTTCG	100	TGCGTTCCTCAGTCCAGATCATGGCATAATGTCAATAAACTATAACACCCCAGAATAAATCCTGGGGCTACCTTTCTAAAGACTTTTTACCACGTTCCAAACTGACGCTGACTCGAGATAACTGAAAAGTGCATACCCCCCTTCTCTCTTGCGAAAGAAACGGAATACTGAATTTCATCACTCAATACTGGTTTCA	100
Salamandra salamandra	Long	AAAAAGACGAGAAAGATATTCATTTTTGAATCCATGATTTAGTTTGTCTACTATGAATAGATGCGAATAAAACCACATTCAATTACATTTCGACAAAAAAAGGGAATTAAAAGTTTTCTCCTTCAACTTGATTTAAAGCGAGTTGGTTTTCTTTTGAAAAAAAGAACCTCAACTAATCACTCATTATCTGCTCCATCTCCCCCTCTTCATCCCTAACCCTATTTTTATATCACTTTTTAGATGATATAAAAAGACAAGGAAATGAATTAAAAAAAGAAAAATAGAACAAGAAAATACTATTGATTCTCAAACAGGCATACTCTACAAAGTAGAGTGCAATGTGCGTTCAAAGATTCGATGATTCACTGAGTTCTGCAATTCACATTACGTATCGCATTTCG	100	TGCGTTCCTCAGTCCAGATCATGGCATAATGTCAATAAACTATAACACCCCAGAATAAATCCTGGGGCTACCTTTCTAAAGACTTTTTACCACGTTCCAAACTGACGCTGACTCGAGATAACTGAAAAGTGCATACCCCCCTTCTCTCTTGCGAAAGAAACGGAATACTGAATTTCATCACTCAATACTGGTTTCA	100
Salamandra atra				TGCGTTCCTCAGTCCAGATCATGGCATAATGTCAATAAACTATAACACCCCAGAATAAATCCTGGGGCTACCTTTCTAAAGACTTTTTACCACGTTCCAAACTGACGCTGACTCGAGATAACTGAAAAGTGCATACCCCCCTTCTCTCTTGCGAAAGAAACGGAATACTGAATTTCATCACTCAATACTGGTTTCA	100
Salamandra atra	Short	TCTTGAAATTTAAATCACCTTAGTTAAATGCATATAGGACTACTTGGCAAATGACTTAAAAACACAAGGTTACAACAAGAAAATACTATTGATTCTCAAACAGGCATACTCTACAAAGTAGAGTGCAATGTGCGTTCAAA	100		
Ambystoma maculatum	Short	AAAAAGAAAAA**GG**GAACAAGAAAATACTATTGATTCTCAAACA**T**GCATACTCTACAAA	95		
Salamandra salamandra	Short	TCTTGAAATTTAAATCACCTTAGTTAAATGCATATAGGACTACTTGGCAAATGACTTAAAAACACAAGGTTACAACAAGAAAATACTATTGATTCTCAAACAGGCATACTCTACAAAGTAGAGTGCAATGTGCGTTCAAA	100		
Salamandra salamandra				TGCGTTCCTCAGTCCAGATCATGGCATAATGTCAATAAACTATAACACCCCAGAATAAATCCTGGGGCTACCTTTCTAAAGACTTTTTACCACGTTCCAAACTGACGCTGACTCGAGATAACTGAAAAGTGCATACCCCCCTTCTCTCTTGCGAAAGAAACGGAATACTGAATTTCATCACTCAATACTGGTTTCA	100
Paramesotriton deloustali	Short	TCTTGAAATTTAAATCACCTTAGTTAAATGCATATAGGACTACTTGGCAAATGACTTAAAAACACAAGGTTACAACAAGAAAATACTATTGATTCTCAAACAGGCATACTCTACAAAGTAGAGTGCAATGTGCGTTCAAA	100		
Salamandra salamandra	Short	TCTTGAAATTTAAATCACCTTAGTTAAATGCATATAGGACTACTTGGCAAATGACTTAAAAACACAAGGTTACAACAAGAAAATACTATTGATTCTCAAACAGGCATACTCTACAAAGTAGAGTGCAATGTGCGTTCAAA	100		
Pleurodeles nebulosus	Short	GCTCCATCTCCCCCTCTTCATCCCTAACCCTATTTTTATATCAC**YW**TTTAGATGATATAAAAAGACAAGGAAATGAATTAAAAAAAGAAAAATAGAACAAGAAAATACTATTGATTCTCAAA**Y**AGGCATACTCTACAAAGTAGAGTGCAATGTGCGTTC	98		
Paramesotriton hongkongensis	Short	TCTTGAAATTTAAATCACCTTAGTTAAATGCATATAGGACTACTTGGCAAATGACTTAAAAACACAAGGTTACAACAAGAAAATACTATTGATTCTCAAACAGGCATACTCTACAAAGTAGAGTGCAATGTGCGTTCAAA	100		
